# Blood Biomarkers Associated with Cognitive Decline in Early Stage and Drug-Naive Parkinson’s Disease Patients

**DOI:** 10.1371/journal.pone.0142582

**Published:** 2015-11-13

**Authors:** Jose A. Santiago, Judith A. Potashkin

**Affiliations:** The Cellular and Molecular Pharmacology Department, The Chicago Medical School, Rosalind Franklin University of Medicine and Science, North Chicago, IL, United States of America; Semmelweis University, HUNGARY

## Abstract

Early diagnosis of Parkinson’s disease (PD) continues to be a major challenge in the field. The lack of a robust biomarker to detect early stage PD patients has considerably slowed the progress toward the development of potential therapeutic agents. We have previously evaluated several RNA biomarkers in whole blood from participants enrolled in two independent clinical studies. In these studies, PD patients were medicated, thus, expression of these biomarkers in *de novo* patients remains unknown. To this end, we tested ten RNA biomarkers in blood samples from 99 untreated PD patients and 101 HC nested in the cross-sectional Parkinson’s Progression Markers Initiative by quantitative real-time PCR. One biomarker out of ten, *COPZ1* trended toward significance (nominal p = 0.009) when adjusting for age, sex, and educational level. Further, *COPZ1*, *EFTUD2* and *PTBP1* mRNAs correlated with clinical features in PD patients including the Hoehn and Yahr scale, Movement Disorder Society revision of Unified Parkinson’s Disease Rating Scale (MDS-UPDRS) and Montreal Cognitive Assessment (MoCA) score. Levels of *EFTUD2* and *PTBP1* were significantly higher in cognitively normal PD patients (PD-CN) compared to cognitively impaired PD patients (PD-MCI). Interestingly, blood glucose levels were significantly higher in PD and PD-MCI patients (≥ 100 mg/dL, pre-diabetes) compared to HC. Collectively, we report the association of three RNA biomarkers, *COPZ1*, *EFTUD2* and *PTBP1* with clinical features including cognitive decline in early drug-naïve PD patients. Further, our results show that drug-naïve PD and PD-MCI patients have glucose levels characteristic of pre-diabetes patients, suggesting that impaired glucose metabolism is an early event in PD. Evaluation of these potential biomarkers in a larger longitudinal study is warranted.

## Introduction

Parkinson’s disease (PD) is a debilitating neurodegenerative disease characterized by the progressive loss of dopaminergic neurons in the substantia nigra pars compacta. Although PD has been largely recognized as a classical movement disorder, non-motor symptoms including constipation, hyposmia, depression, sleep behavior disorders are now being recognized as early prodromal symptoms of the disease [1, 2]. Diagnosis of PD remains challenging and there is a 20–30% misdiagnosis rate with atypical parkinsonian disorders [3]. To date, diagnosis of PD is based on the assessment of clinical motor symptoms. The lack of a robust biomarker with high sensitivity and specificity has greatly hampered the validation of potential therapeutics.

Blood biomarkers are emerging as promising diagnostic tools for PD. Altered expression of several RNAs [4–8]and microRNAs [9, 10] have been identified in medicated PD patients, although, very few RNA biomarkers have been replicated in samples from independent clinical studies. For instance, a molecular signature in blood composed of 13 splice variants, including *C5ORF4*, *COPZ1*, *MACF1*, *WLS*, *PRG3*, *ZNF160*, *EFTUD2*, *MAP4K1*, *MPP1*, *PKM2*, *SLC14A1-s*, *SLC14A1-l* and *ZNF134*, was useful to distinguish PD patients from atypical parkinsonian disorders and healthy controls (HC) nested in the Diagnostic and Prognostic Biomarkers for Parkinson’s Disease (PROBE) with 90% sensitivity and 94% specificity [11]. Seven out of the 13 biomarkers including *C5ORF4*, *COPZ1*, *MACF1*, *WLS*, *PRG3*, *ZNF160* and *EFTUD2*, were replicated in blood of PD patients and HC nested in the Harvard Biomarker Study (HBS) with 78% sensitivity and 90% specificity [12]. Using network-based approaches, other potential RNA biomarkers including *APP*, *SOD2*, *HNF4A* and *PTBP1* were identified as useful for distinguishing PD patients from HC [13–16]. Among this group, *HNF4A* and *PTBP1* were able to distinguish PD patients from HC with 90% sensitivity and 80% specificity.

Despite this progress, PD patients enrolled in all of these studies were already medicated. More recently, one study identified several candidate biomarkers in untreated PD patients, but these have yet to be validated [17]. To test whether markers that have been replicated in independent clinical trials are also useful for diagnosing unmedicated PD patients, we evaluated ten RNA biomarkers in early-stage drug-naïve PD patients and HC enrolled in the Parkinson’s Progression Markers Initiative (PPMI) [18]. We identified three potential blood biomarkers, *COPZ1*, *EFTUD2* and *PTBP1* mRNAs that correlated with clinical features in the largest to date cohort of untreated PD patients. Further, *EFTUD2* and *PTBP1* may be useful to distinguish PD patients with cognitive impairment. Evaluation of these potential biomarkers in a larger longitudinal study and patients at risk of PD is warranted.

## Material and Methods

### Study Participants

PPMI is an ongoing 5-year observational, longitudinal, and multi-center study of drug-naïve patients with early stage PD (n = 423) and HC (n = 196) recruited from 32 clinical sites in Australia, United States of America (USA) and Europe [18]. A power analysis based on each biomarker from our previous studies [12–15], indicated that a minimum fold change of 1 between PD and controls could be detected with a 95% power using 100 samples per group. Thus, we selected a total of 200 participants, 99 PD patients and 101 age and sex matched HC nested in the PPMI study PD patients that had a dopamine transporter deficit assessed by DaTscan imaging. PD patients with a Hoehn and Yahr stage I or II were chosen for this study so that we could relate the results to our earlier studies on medicated patients at these stages of the disease. Healthy individuals were cognitively normal, free of neurological disorder, and with no detectable dopamine transporter deficit. Among the 99 PD patients, we selected 35 patients with cognitive impairment as defined by a Montreal Cognitive Assessment (MoCA) score of less than 26. Demographic and clinical characteristics about the study participants are shown in Table 1. The Institutional Review Boards of Rosalind Franklin University of Medicine and Science and all sites participating in the PPMI study approved the study. Written informed consent was obtained from all participants before inclusion in the study. All participants were evaluated for clinical features by investigators at each participant site. More information about study participants have been described elsewhere [19] and at the PPMI website (http://www.ppmi-info.org/).

**Table 1:** Comparison of demographic and clinical characteristics between PD patients and HC.

Characteristic	HC (n = 101)	PD (n = 99)	P value ^a^
Age, mean (SD) [95% CI], y	61 (10) [59–63]	63 (9) [61–65]	0.19
Female/male, No. (%male)	45/56 (55.4)	49/50 (50.5)	0.57^b^
Education, mean (SD) [95% CI], y	16.2 (2.9) [15.6–16.8]	15.1 (3.2) [14.4–15.7]	0.02
Disease duration, media (range), months	n/a	4 (1–36)	n/a
Hoehn and Yahr stage, mean (SD)	0.009 (0.09)	1.44 (0.50)	<0.001
MDS-UPDRS total	4.87 (4.41)	31.79 (12.41)	<0.001
MDS-UPDRS part I	0.60 (1.06)	1.28 (1.42)	<0.001
MDS-UPDRS part I–patient questionnaire	2.40 (2.30)	4.71 (3.36)	<0.001
MDS-UPDRS part II-patient questionnaire	0.45 (0.99)	5.82 (4.13)	<0.001
MDS- UPDRS part III-patient questionnaire	1.43 (2.30)	19.98 (8.56)	<0.001
MoCA, mean (SD) [95% CI]	28.23 (1.07) [28.02–28.44]	25.98 (2.53) [25.48–26.48]	<0.001
Glucose levels in blood	97.58 (14.13) [94.80–100.4]	101.6 (14.44) [98.75–104.5]	<0.01
UPSIT score, mean (SD)[95%CI]	34.00 (4.86) [33.04–34.96]	21.08 (8.12) [19.46–22.70]	<0.0001
GDS score, mean (SD) [95% CI]	1.27 (1.89) [0.89–1.64]	2.15 (2.48) [1.64–2.67]	<0.007
SCOPAmean (SD) [95% CI]	5.86 (3.28) [5.21–6.51]	9.27 (5.40) [8.16–10.38]	<0.0001

Demographic and clinical information of PPMI participants. Abbreviations: CI = 95% confidence interval; GDS = Geriatric Depression Scale; HC = healthy controls; MoCA = Montreal Cognitive Assessment; MDS-UPDRS = Movement Disorder Society-sponsored revision of the Unified Parkinson’s Disease Rating Scale; PD = Parkinson’s disease; SCOPA = Scale for Outcomes in Parkinson’s disease for Autonomic Symptoms SD = standard deviation; y = years. UPSIT = University of Pennsylvania Smell Identification Test.

^a^ Based on a Student t-test.

^b^ Based on chi-square test (X^2^).

### Blood sample collection and handling

Baseline whole blood collection was performed at each participant site as described in the PPMI biologics manual (http://www.ppmi-info.org/). Blood samples were collected in the morning between 8am-10am, and patients were asked to fast before blood collection. Blood was collected in PAXgene blood RNA tubes following the study protocol. Briefly, PAXgene tubes containing blood were immediately inverted gently 8–10 times to mix the samples. Tubes were placed upright and incubated for 24 hrs at room temperature before freezing. After the 24 hrs incubation period, PAXgene blood tubes were stored at -80^0^ F until shipment. Frozen samples were sent to the PPMI Biorepository Core laboratories for RNA extraction. Blinded frozen samples (n = 200) were shipped in dry ice to Rosalind Franklin University of Medicine for the studies described herein.

### Quantitative Polymerase Chain Reaction Assays

Samples with RNA integrity values **>**7.0 and absorbance 260/280 between 1.2 and 3.0 were used in this study. One microgram of RNA was reverse transcribed into cDNA using a mix of random hexamer primers (High Capacity cDNA Synthesis Kit, Life Technologies, USA). Quantitative polymerase chain reaction assays (qRT-PCR) were performed using the DNA engine Opticon 2 Analyzer (Bio-Rad Life Sciences, Hercules, CA, USA). Each 25 microliters reaction contained Power SYBR Green (Life Technologies, USA) and primers at a concentration of 0.05 mM. Samples were run in triplicates. Amplification conditions and primer sequences have been published elsewhere [12–15]. All the data collected in this study will be publicly available at (http://www.ppmi-info.org/).

### Statistical Analysis

Statistical analysis was performed using STATISTICA 12 (StatSoft, OK, USA) and GraphPad Prism version 5 (GraphPad Software, Inc., CA, USA). A Student-t-test (unpaired, two tailed) was used to assess the differences between two groups and a chi-square test was used to analyze categorical data. Pearson correlation was performed for all correlations. A Pearson correlation analysis of the RIN values and expression of each biomarker showed no significant associations (p = 0.9). The relative abundance of each biomarker was independently assessed using a general linear regression model adjusting for age, sex, and educational level. A Bonferroni adjustment was used to correct for multiple comparisons and a p-value of 0.005 or less was considered significant.

In addition, we evaluated the expression of each RNA biomarker independently in PD patients with cognitive decline compared to cognitively normal PD patients. For this analysis, we used a general linear regression model adjusting for age, gender, education, Hoehn and Yahr, and MDS-UPDRS. A receiver operating characteristic curve (ROC) analysis was performed to determine the diagnostic accuracy of each biomarker. A nominal p-value of 0.05 or less was considered significant

## Results

### Demographic characteristics of study participants

There were no significant differences in mean age and sex distribution between PD patients and HC. However, PD patients had a small but significant difference in the number of years they attended school compared to HC (Table 1). As expected, comparisons of clinical features showed significant differences in Hoehn and Yahr stage, Movement Disorder Society-sponsored revision of the Unified Parkinson’s Disease Rating Scale (MDS-UPDRS), MoCA score, University of Pennsylvania Smell Identification Test (UPSIT), Geriatric Depression Scale (GDS) and Scale for Outcomes in Parkinson’s disease for Autonomic Symptoms (SCOPA) scores between PD patients and controls (Table 1). We also compared the blood glucose levels in PD patients and controls. Mean blood glucose level was within the normal range in HC (<100 mg/dL) but was significantly higher than normal in the PD group (≥ 100 mg/dL) (Table 1).

### Evaluation of RNA biomarkers in blood of PPMI study participants

**Fig 1:**
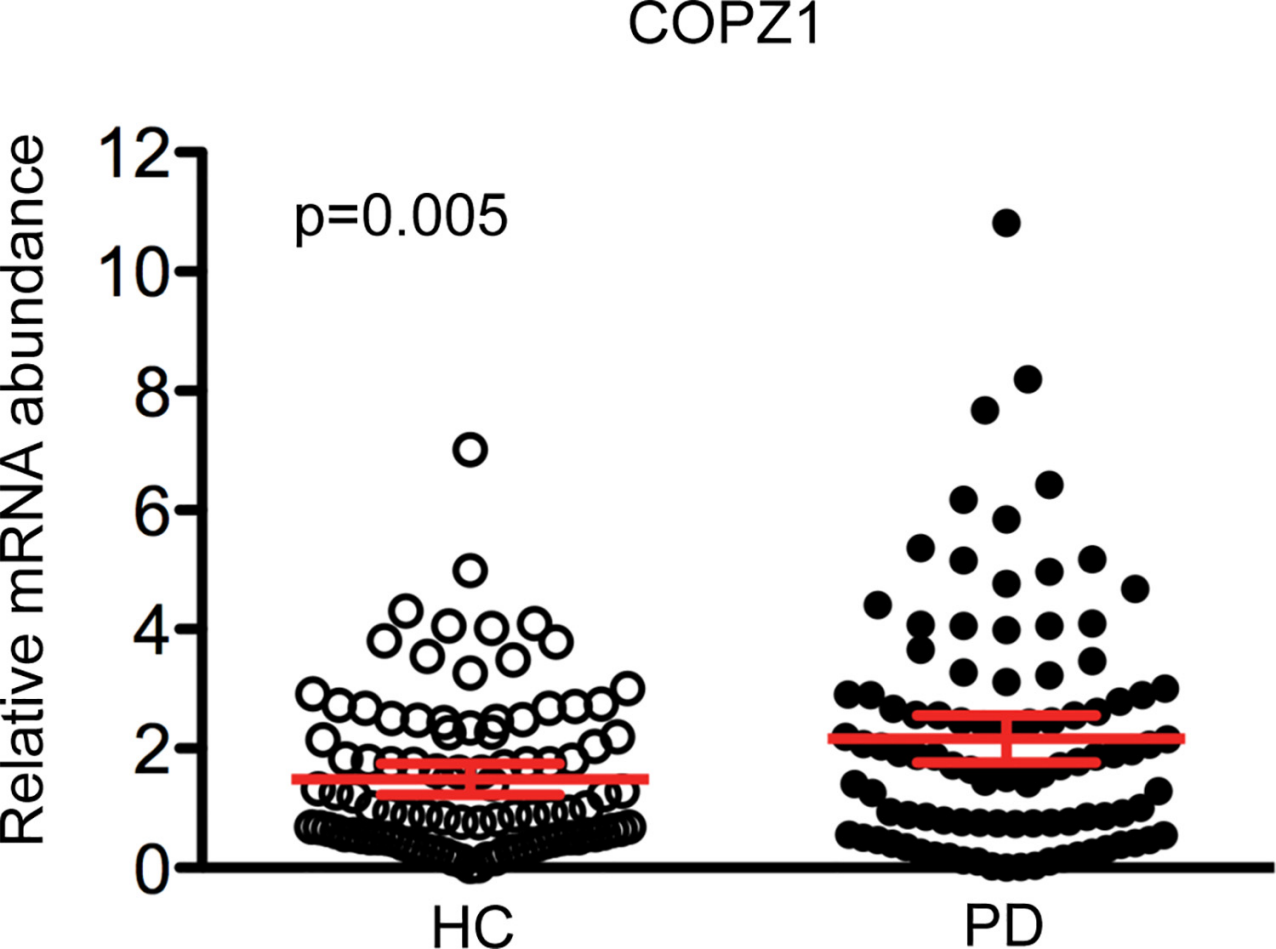
Relative abundance of *COPZ1* mRNA in the PPMI study. Relative abundance of *COPZ1* in PD patients (black circles) compared to HC (white circles). Relative abundance of *COPZ1* was calculated using *GAPDH* mRNA as a reference gene. Error bars represent the 95% confidence interval. A p-value of less than 0.005 was regarded as significant based on a Student t-test (two-tailed).

A total of ten RNA biomarkers were tested in the PPMI cohort by quantitative PCR assays. No significant difference was observed in the expression of 9 of the markers, including *EFTUD2*, *PTBP1*, *C5ORF4*, *APP*, *SOD2*, *HNF4A*, *WL*S, *ZNF160* and *MACF1*, between PD patients and controls in the univariate analysis (S1 Table). Interestingly however, a small but significant difference in the expression of *COPZ1* was identified in the blood of PD patients compared to HC. Specifically, the relative abundance of *COPZ1* (p = 0.005) was significantly up-regulated in PD patients compared to HC, although overlap in expression levels between the two groups was observed (Fig 1, S1 Table). In addition, the analysis was performed using a general linear regression model adjusting for confounding variables including age, sex and educational level. Relative abundance of *COPZ1* trended toward significance in PD patients compared to HC after adjusting for confounding variables (ß = 0.18, nominal p = 0.009).

Pearson correlation analysis was performed to determine whether the relative abundance of each RNA biomarker was independent of age, gender and educational level and clinical outcome variables. The relative abundance of *COPZ1* was independent of age, gender and education (S2 Table). Interestingly, the relative abundance of three biomarkers, *COPZ1*, *EFTUD2* and *PTBP1* correlated with measures of disease severity in PD patients. For instance, *COPZ1* correlated positively with the Hoehn and Yahr scale, MDS-UPDRS total, and MDS-UPDRS parts II and III. Relative abundance of *EFTUD2* correlated negatively with age, Hoehn and Yahr scale, MDS-UPDRS total, MDS-UPDRS parts I, II and III and correlated positively with MoCA (S2 Table). Relative abundance of *PTBP1* correlated negatively with MDS-UPDRS parts I and positively with MoCA. In addition, *COPZ1* and *EFTUD2* correlated negatively with the UPSIT and GDS scores, respectively.

### *EFTUD2* and *PTBP1* are associated with cognitive decline in PD

Since cognitive decline is common in PD, we evaluated the clinical differences between cognitively normal PD patients (PD-CN) and PD patients with mild cognitive impairment (PD-MCI). Cognitive impairment was defined using the recommended MoCA cutoff greater than 26 as shown previously [20, 21]. HC were excluded from this analysis. There were no significant differences in age, educational level, disease duration, and disease severity scales including the Hoehn and Yahr and MDS-UPDRS between PD-CN and PD-MCI (Table 2). However, there were a significant higher number of male participants in the PD-MCI group (Table 2). Interestingly, mean blood glucose levels are significantly higher than normal in PD-MCI (mean (SD) 95% CI: 105.8 (15.27) [100.6–111.0]) compared to PD-CN (Table 2).

**Table 2:** Comparison of demographic and clinical characteristics of cognitively normal PD patients (PD-CN) and cognitively impaired PD patients (PD-MCI).

Characteristic	PD-CN (n = 64)	PD-MCI (n = 35)	P value^a^
Age, mean (SD)[95% CI], y	62 (8) [60–64]	65 (9) [62–68]	0.17
Male/Female, No. (%male)	23/41 (56)	27/8 (338)	0.0001^b^
Education, mean (SD) [95% CI],y	15.02 (3.44) [14.16–15.87]	15.14 (2.68) [14.22–16.06]	0.85
Disease duration, median (range), months	4 (1–36)	5 (1–23)	0.80
Hoehn and Yahr stage, mean (SD)	1.44 (0.50)	1.46 (0.50)	0.85
MDS-UPDRS total	31.31 (12.34)	32.66 (12.68)	0.61
MDS-UPDRS part I	1.31 (1.29)	1.23 (1.64)	0.78
MDS-UPDRS part I-patient questionnaire	4.83 (3.38)	4.49 (3.35)	0.63
MDS-UPDRS part II-patient questionnaire	6.17 (4.32)	5.17 (3.73)	0.25
MDS- UPDRS part III-patient questionnaire	19.00 (7.93)	21.77 (9.46)	0.12
MoCA, mean (SD) [95% CI]	27.53 (1.08) [27.26–27.80]	23.14 (1.85) [22.51–23.78]	<0.0001
UPSIT score,mean (SD) [95% CI]	21.97 (8.00) [19.97–23.97]	19.46 (8.18) [16.65–22.27]	0.14
GDS score,mean (SD) [95% CI]	2.00 (2.24) [1.42–2.59]	2.42 (2.87) [1.41–3.44]	0.43
SCOPA,mean (SD) [95% CI]	9.46 (5.60) [8.02–10.91]	8.91 (5.08) [7.11–10.71]	0.64
Blood glucose levels,mean (SD) [95% CI]	99.34 (13.55) [95.96–102.7]	105.8 (15.27) [100.6–111.0]	0.03

Demographic and clinical characteristics of cognitively normal PD patients (PD-CN) and cognitively impaired PD patients (PD-MCI). Abbreviations: CI = 95% confidence interval; GDS = Geriatric Depression Scale; HC = healthy controls; MoCA = Montreal Cognitive Assessment; MDS-UPDRS = Movement Disorder Society-sponsored revision of the Unified Parkinson’s Disease Rating Scale; PD = Parkinson’s disease; SCOPA = Scale for Outcomes in Parkinson’s disease for Autonomic Symptoms SD = standard deviation; y = years. UPSIT = University of Pennsylvania Smell Identification Test.

^a^ Based on Student t-test.

^b^ Based on chi-square test (X^2^).

**Fig 2:**
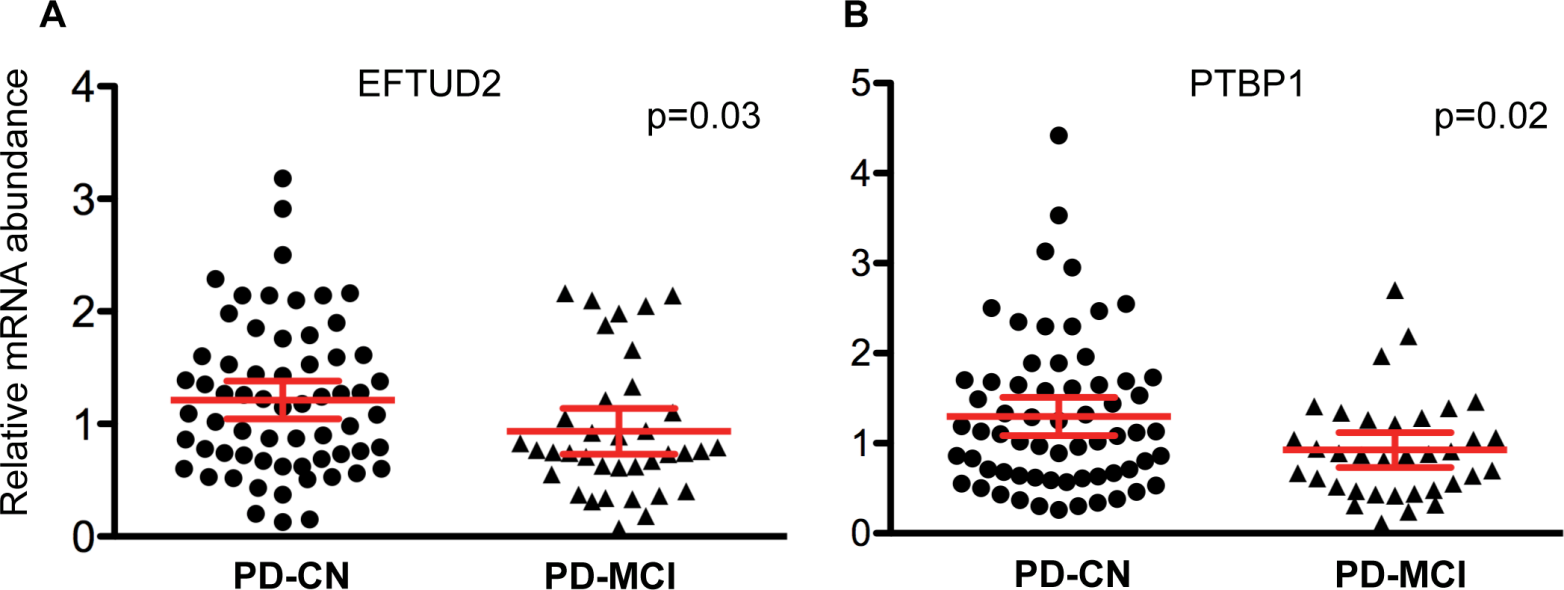
*EFTUD2* and *PTBP1* mRNAs as biomarkers for cognitive decline in PD. A. Relative abundance of *EFTUD2* in PD patients with normal cognition (circles) compared to PD patients with mild cognitive impairment (triangles). B. Relative abundance of *PTBP1* in PD patients with normal cognition (circles) compared to PD patients with mild cognitive impairment (triangles). Error bars represent the 95% confidence interval. A p-value of less than 0.05 was regarded as significant based on a Student t-test (two-tailed). PD-CN is cognitively normal PD patients, PD-MCI is PD patients with mild cognitive impairment.

Given the significant correlation between *EFTUD2* and *PTBP1* with MoCA score we evaluated their capability to distinguish PD-CN from PD-MCI patients. Expression of *EFTUD2* was significantly up-regulated in PD-CN (mean (SD) [95% CI] p-value, 1.21 (0.66) [1.04–1.38] p = 0.04) compared to PD-MCI (0.93 (0.60) [0.72–1.14]) (Fig 2A). In parallel, expression of *PTBP1* was significantly up-regulated in PD-CN (1.30 (0.84) [1.09–1.51] p = 0.02) compared to PD-MCI (0.93 (0.56) [0.73–1.12]) (Fig 2B). Pearson correlation analysis showed that *EFTUD2* expression was independent of age, gender and years of education (p> 0.5). Similarly, *PTBP1* expression did not correlate with age (p = 0.8), and gender (p = 0.19), but it showed a significant but weak correlation with years of education (r = -0.20, p = 0.05). This analysis was repeated using a general linear regression model adjusting for confounding variables including age, sex, educational level, Hoehn and Yahr, disease duration, and MDS-UPDRS. Both biomarkers, *EFTUD2* (ß = 0.27, p = 0.01) and *PTBP1* (ß = 0.22, p = 0.04) reached statistical significance. ROC analysis for *EFTUD2* and *PTBP1* resulted in AUC values of 0.64 and 0.63, respectively.

Pearson correlation analysis showed significant and positive correlations among COPZ1 *EFTUD2* and *PTBP1*. For example, *COPZ1* correlated with *EFTUD2* (r = 0.14, p = 0.04) and *PTBP1* (r = 0.32, p = 0.0001). The highest correlation value was observed between *EFTUD2* and *PTBP1* (r = 0.61, p = 0.001).

## Discussion

Validation of candidate biomarkers for PD in several independent clinical studies is an important step toward their effective translation to the clinic. One crucial step in this process is the evaluation of biomarkers in drug-naïve patients to ensure that commonly prescribed medications for PD do not have an effect in their expression. Here we tested ten blood RNA biomarkers that were evaluated previously in two independent clinical studies, in blood of untreated PD patients and controls enrolled in the PPMI study.

Three candidate blood biomarkers, *COPZ1*, *EFTUD2* and *PTBP1*, correlated with clinical features in early stage drug-naïve PD patients suggesting they may be useful for patient stratification according to disease severity measures. Specifically, *COPZ1* significantly correlated with Hoehn and Yahr scale, MDS-UPDRS total, MDS-UPDRS parts II and III and UPSIT score. Similarly, *EFTUD2* showed significant correlations with Hoehn and Yahr, MDS- UPDRS total, MDS- UPDRS parts I, II, and III, MoCA and GDS scores. In addition, *PTBP1* significantly correlated with MDS-UPDRS part I, and MoCA score. These findings suggest that *COPZ1*, *EFTUD2* and *PTBP1* may be useful biomarkers to classify PD patients with regards to symptoms. Nonetheless, these results need to be taken with caution since the biomarker correlations with clinical features were relatively weak. Thus, evaluation of these potential biomarkers in a larger longitudinal study will be required to assess their prognostic utility.

One biomarker, *COPZ1* trended toward significance (nominal p = 0.009) after adjusting for age, sex and educational level. It is important to note, that we observed overlap in the relative abundance of *COPZ1* between PD and controls and low diagnostic capacity. In addition, we previously reported that the relative abundance of *COPZ1* was up-regulated in blood of medicated PD patients enrolled in two independent clinical studies [11, 12]. It should be noted that the magnitude of the change in *COPZ1* expression was greater in our earlier reports using medicated PD patients compared to unmedicated patients in this report (5-fold vs 1.5-fold). Together these results suggest that medication has an impact on the expression of *COPZ1*.

Cognitive decline is a common non-motor aspect of PD that frequently progresses to dementia. In fact, up to 80% of PD patients with mild cognitive impairment will eventually develop dementia [22]. Thus, biomarkers that predict changes in cognitive abilities are essential for the development of therapeutic strategies for PD [23]. Several potential biomarkers of cognitive decline have been identified. For example, CSF levels of amyloid beta peptides (Aß), but not total tau, were associated with cognitive impairment in untreated PD patients enrolled in the Norwegian ParkWest study[24]. Contrary to these findings, Aß peptides, total tau and phosphorylated tau (p-tau) did not correlate with cognitive measures in untreated patients in a larger cohort of patients enrolled in Deprenyl and Tocopherol Antioxidative Therapy of Parkinsonism (DATATOP) study. However, higher p-tau and p-tau/Aß42 levels in the CSF predicted cognitive decline in PD patients after levodopa treatment [25]. In addition, serum levels of the insulin growth factor 1 (IGF-1) and epidermal growth factor (EGF) are associated with cognitive decline in early stage and drug-naïve patients with PD [26–30]. Approximately 10% of PD patients met the criteria for cognitive impairment in the PPMI study [21]. In this study, relative abundance of *EFTUD2* and *PTBP1* was significantly up-regulated in PD-CN compared to PD-MCI independently of age, gender, disease duration, and disease severity measures. To the best of our knowledge, these are the first blood RNA biomarkers that have been associated with cognitive decline in PD. Future studies in larger and well-characterized longitudinal studies will be important to assess their predictive performance in PD patients.

The biomarkers examined in this study are related to biological processes associated with PD. For example, COPZ1 encodes a subunit of the cytoplasmic coatomer complex, which is involved in autophagy and protein trafficking. EFTUD2, elongation factor Tu GTP binding domain containing 2, which encodes the splicing factor U5-116 kD, is involved in RNA splicing via the spliceosome. In this regard, the involvement of aberrant RNA splicing in blood of PD has been noted in numerous studies [7, 11, 12, 15], reviewed in [31]. In this regard, a recent study identified a network of dysregulated splicing factors in blood of PD patients supporting the idea that splicing may be both inefficient and dysregulated in PD [15].

The polyprimidine tract binding protein1 (PTBP1) belongs to the family of heterogeneous nuclear ribonucleoproteins (hnRNPs) that are involved in pre-mRNA processing, metabolism and transport. PTBP1 plays a pivotal role in the mRNA stabilization and translation of insulin in the pancreas and it has been associated with diabetes [32]. Thus, altered expression of *PTBP1* may be an important indicator of impaired glucose and insulin signaling in de novo PD patients.

In this context, mounting evidence indicates that impaired glucose metabolism, insulin signaling and diabetes are associated with PD [13, 33, 34]. Treatment with levodopa exacerbates glucose intolerance in PD patients [35, 36], but abnormal blood glucose levels in untreated PD patients have not yet been reported. According to the American Diabetes Association (http://www.diabetes.org/diabetes-basics/diagnosis/), fasting blood glucose levels ≥100 mg/dL are characteristic of subjects with pre-diabetes. Accordingly, blood glucose levels were significantly higher than normal in PD (101.6 mg/dL) and PD-MCI (105.8 mg/dL) patients. Thus, we report for the first time that drug-naïve PD and PD-MCI patients have glucose levels characteristic of pre-diabetes patients, suggesting that impaired glucose metabolism is an early event in PD. These results are representative of a subset of participants enrolled in PPMI study and therefore warrant further investigation.

Collectively, we report the association of three RNA biomarkers with clinical features including cognitive decline in early drug-naïve PD patients. Nevertheless, the diagnostic performance of these biomarkers is low and below the ideal characteristics for a diagnostic biomarker. There are several important factors that need to be considered when interpreting the results from this study and its discrepancies from our previous studies. First, our biomarkers were tested previously in very homogenous clinical studies in the USA [11, 12, 14, 15], where most of the PD patients were medicated. PPMI is an international multicenter study of drug-naïve PD patients and HC recruited from 32 different clinical sites including Europe, Australia and USA. Thus, other factors related to the diverse ethnic backgrounds, diet, and epigenetic changes, for example, may explain the discrepancies in biomarker expression observed in this study. In addition, even with the rigorous standard procedures for sample collection, processing and storage followed by the PPMI study, there is a potential for pre-analytical variability introduced by the different clinical sites [37].

Unbiased approaches are needed to identify highly sensitive and specific diagnostic biomarkers for the identification of early stage and pre-symptomatic PD patients [38]. We foresee that emerging high-throughput technologies combined with network analyses using early stage untreated and/or pre-symptomatic PD patients will be instrumental in the discovery of new potential biomarkers [16]. Evaluation of these biomarkers in larger longitudinal studies is needed to test further their prognostic and diagnostic utility.

## Supporting Information

S1 Table Relative expression levels of blood biomarkers in HC and patients with PD. ^a^ Assessed by Student t-test (two-tailed). CI is the 95% confidence interval. A p-value of 0.005 or less was considered significant.
(DOC)

S2 Table Pearson (r) correlation of blood biomarkers with clinical outcomes. Pearson correlation of blood biomarkers with clinical outcomes. Pearson correlation (r) is shown for each of the biomarkers and the clinical outcome variables. P-values for significant correlations are shown in parenthesis. Abbreviations: GDS = Geriatric Depression Scale; HC = healthy controls; MoCA = Montreal Cognitive Assessment; MDS-UPDRS = Movement Disorder Society-sponsored revision of the Unified Parkinson’s Disease Rating Scale; PD = Parkinson’s disease; SCOPA = Scale for Outcomes in Parkinson’s disease for Autonomic Symptoms; y = years. UPSIT = University of Pennsylvania Smell Identification Test
(DOC)
