# Supplementary material for: Blood Biomarkers Associated with Cognitive Decline in Early Stage and Drug-Naive Parkinson’s Disease Patients
Source: PLoS One. 2015 Nov 13;10(11):e0142582. doi: 10.1371/journal.pone.0142582 (PMC4643881; doi:10.1371/journal.pone.0142582)
Supplement: S1 Table — a Assessed by Student t-test (two-tailed). CI is the 95% confidence interval. A p-value of 0.005 or less was considered significant. (DOC) [file pone.0142582.s001.doc]

**Table S1. Relative expression levels of blood biomarkers in HC and patients with PD.**

| **Biomarker** | **HC (n=101)**  **mean, (SD)**  **[95% CI]** | **PD (n=99)**  **mean, (SD) [95% CI]** | **P-value^a^** |
| --- | --- | --- | --- |
| COPZ1 | 1.49 (1.29)  [1.22-1.74] | 2.16 (1.90)  [1.76-2.56] | 0.005 |
| EFTUD2 | 1.39 (1.12)  [1.17-1.61] | 1.11 (0.65)  [0.98-1.24] | 0.03 |
| PTBP1 | 1.24 (0.87)  [1.06-1.41] | 1.13 (0.70)  [0.99-1.27] | 0.33 |
| C5ORF4 | 1.35 (1.14)  [1.13-1.58] | 1.44 (1.26)  [1.19-1.69 | 0.62 |
| APP | 1.85 (2.84)  [1.26-2.45] | 2.02 (2.74)  [1.45-2.59] | 0.68 |
| SOD2 | 1.29 (0.88)  [1.11-1.47] | 1.26 (1.01)  [1.05-1.50] | 0.84 |
| HNF4A | 1.84 (2.20)  [1.40-2.28] | 1.85 (2.36)  [1.36-2.33] | 0.99 |
| WLS | 1.76 (1.84)  [1.39-2.13] | 1.85 (1.78)  [1.49-2.21] | 0.73 |
| ZNF160 | 1.57 (1.96)  [1.18-1.96] | 1.47 (1.96)  [1.13-1.81] | 0.69 |
| MACF1 | 1.25 (0.80)  [1.09-1.41] | 1.27 (0.83)  [1.10-1.44] | 0.87 |

^a^ Assessed by Student t-test.

CI is the 95% confidence interval.
