# Supplementary material for: Blood Biomarkers Associated with Cognitive Decline in Early Stage and Drug-Naive Parkinson’s Disease Patients
Source: PLoS One. 2015 Nov 13;10(11):e0142582. doi: 10.1371/journal.pone.0142582 (PMC4643881; doi:10.1371/journal.pone.0142582)
Supplement: S2 Table — Pearson correlation of blood biomarkers with clinical outcomes. Pearson correlation (r) is shown for each of the biomarkers and the clinical outcome variables. P-values for significant correlations are shown in parenthesis. Abbreviations: GDS = Geriatric Depression Scale; HC = healthy controls; MoCA = Montreal Cognitive Assessment; MDS-UPDRS = Movement Disorder Society-sponsored revision of the Unified Parkinson’s Disease Rating Scale; PD = Parkinson’s disease; SCOPA = Scale for Outcomes in Parkinson’s disease for Autonomic Symptoms; y = years. UPSIT = University of Pennsylvania Smell Identification Test (DOC) [file pone.0142582.s002.doc]

**Table S2. Pearson correlation of blood biomarkers with clinical outcomes.**

| **Outcome** | **COPZ1**  **r (p-value)** | **EFTUD2**  **r (p-value)** | **PTBP1**  **r (p-value)** |
| --- | --- | --- | --- |
| Gender | 0.13 | 0.01 | 0.12 |
| Age | -0.05 | -0.23 (0.01) | -0.10 |
| Years of education | -0.10 | 0.00 | -0.07 |
| Disease duration | 0.08 | -0.07 | -0.07 |
| Hoehn and Yahr | 0.19 (0.008) | -0.14 (0.04) | -0.03 |
| MDS-UPDRS Total | 0.19 (0.008) | -0.18 (0.01) | -0.09 |
| MDS-UPDRS Part I | 0.06 | -0.06 | -0.05 |
| MDS-UPDRS Part I - Patient Questionnaire | 0.10 | -0.20 (0.004) | -0.17 (0.02) |
| MDS-UPDRS Part II - Patient Questionnaire | 0.18 (0.01) | -0.14 (0.04) | -0.09 |
| MDS-UPDRS Part III - Patient Questionnaire | 0.18 (0.01) | -0.14 (0.04) | -0.04 |
| MoCA score | 0.05 | 0.23 (0.001) | 0.15 (0.03) |
| Glucose levels in CSF | -0.10 | 0.03 | -0.17 (0.03) |
| UPSIT score | -0.19 (0.007) | 0.09 | 0.01 |
| GDS score | 0.04 | -0.14 (0.04) | -0.08 |

**Table S2**. Pearson correlation of blood biomarkers with clinical outcomes. Pearson correlation (r) is shown for each of the biomarkers and the clinical outcome variables. P-values for significant correlations are shown in parenthesis. Abbreviations: GDS = Geriatric Depression Scale; HC = healthy controls; MoCA = Montreal Cognitive Assessment; MDS-UPDRS = Movement Disorder Society-sponsored revision of the Unified Parkinson’s Disease Rating Scale; PD = Parkinson’s disease; SCOPA = Scale for Outcomes in Parkinson’s disease for Autonomic Symptoms; y = years. UPSIT = University of Pennsylvania Smell Identification Test
